# A Study on the Place Attachment of Golf Club Members

**DOI:** 10.3389/fpsyg.2020.00408

**Published:** 2020-04-09

**Authors:** Chun Chen, Shu-Wang Lin, Shih-Yun Hsu, Chi-Hsuan Wu

**Affiliations:** ^1^Changzhou Institute of Industry Technology, Changzhou, China; ^2^Office of Physical Education, Chienkuo Technology University, Changhua, Taiwan; ^3^Department of Business Management, National Taichung University of Science and Technology, Taichung City, Taiwan; ^4^Graduate Institute of Sports and Health Management, National Chung Hsing University, Taichung, Taiwan

**Keywords:** golf, clubs, activities involved, place attachment, leisure sport

## Abstract

The purpose of this study is to explore the members of golf clubs in the central region of Taiwan and find out whether their involvement in activities affects the degree of place attachment and to add the two factors of activity experience and experience value so as to develop a theoretical framework. A questionnaire survey was used to collect 534 samples from golf clubs in central Taiwan for analysis using the following research tools: the Activity Involvement scale, Place Attachment scale, and Likert psychological scale. The results of the study show that (1) activity involvement has a significant positive impact on place attachment, activity experience, and experience value; (2) activity experience has a significant positive impact on experience value; (3) experience value has a significant positive impact on place attachment. This result verifies the theory that activity involvement impacts place attachment. It is suggested that the relevant bodies should strengthen the incentives given in the activities and strengthen the value of the leisure experience so as to facilitate the development of related industries in the future.

## Introduction

With economic growth, the demands of modern people for quality of life are increasing day by day, and they are beginning to pursue a variety of leisure activities (Wang et al., 2012). Leisure activities are considered to improve quality of life and health (Vassiliadis et al., 2013a; Chick et al., 2016; Mehraliyev et al., 2019). There are many kinds of leisure activities, but according to degree of participant involvement, leisure activities can be roughly divided into two types: casual and serious (Scott, 2012). Golf, the game discussed in this study, is a kind of serious leisure activity. Those who engage in serious leisure activities (such as golf) must have certain professional skills (such as with golf technology) and invest a certain amount of time, money, and other resources in the activities they are engaged in. Serious leisure activities are thus usually highly involving activities, and the leisure benefits to the participants are higher than from casual leisure activities. Barbieri and Sotomayor (2013) summarize the differences in benefits between the two types of leisure, including social difficulties that are span difficulties, self-growth, engaging in leisure activities as a career, achieving certain long-term benefits, constructing a self-image, and creating leisure activities.

Sport has gained increasing popularity in Taiwan. This is partly due to the success of many well-known athletes. Some of the more popular forms of sport are basketball, baseball, badminton, and tennis. After Yani Tseng won many international trophies, golf also gained significantly in popularity. However, because golf requires a large area of well-maintained grassland, which is quite scarce in Taiwan, golf remains a very expensive sport. An aging society has become the trend of the future, and as a result, enterprises have increasingly begun to take notice of the value of this market segment and to develop the expertise to meet its product needs, including travel and leisure products (Kim et al., 2015), and golf is a leisure activity that is good for the elderly. Danylchuk et al. (2015) found that more and more women are beginning to join the ranks of golf leisure participants, and as the market of older people expands, the importance of golf increases. Further, some companies are using technology to develop virtual golf courses and to offer a variety of golf activities for consumers to choose from, and so golf-related research is becoming increasingly important (Han et al., 2014).

Involvement means the degree to which an individual perceives a person, thing, or activity as important because of his or her needs, values, and interests (Chiu et al., 2014). Chiu et al. (2014) posit that the higher the level of activity involvement, the greater the willingness to partake in and frequency of activities. The concept of involvement stems from consumer behavior, which involves five elements, namely importance, pleasure, symbolism, risk, and risk consequences (Laurent and Kapferer, 1985; Kyle et al., 2003; Hwang et al., 2005; Mansfeld and McIntosh, 2009; Vassiliadis et al., 2013b). These five elements affect the consumer’s willingness to purchase a product or service and the attitude toward the product or service. Later, the concept of involvement was adopted in tourism and leisure-related fields (Lu et al., 2015) and termed activity involvement, meaning a person’s level of immersion in the activity experience (Prayag and Ryan, 2012), while the introduction of activity involvement are considered to be one of the important variables for predicting tourism or leisure behavior (Prebensen et al., 2012) and attitudes toward a place or activity (Shen et al., 2012). The elements involved in the activity contain three different originals, namely attraction, self-expression, and life center (Funk et al., 2004; Fotiadis and Vassiliadis, 2016); attraction means the attraction of the activity to the person, self-expression means whether people define their own values through the activity, and finally, centrality of life is the impact of the activity on people and their social circles, which coincides with the leisure benefits mentioned by Barbieri and Sotomayor (2013). In other words, as the level of involvement in the activity increases, the leisure benefits that individuals receive from the activity increase, and they are more willing to continue to engage in the activity.

In summary, the research problem is mainly based on golf leisure activities, taking golf course customers as an example to explore the degree of involvement of golf course customers in their activities and to understand their leisure behavior. Based on this research question, the purpose of this study is to explore the relationship between golf course customer activity, activity experience, experience value, and local attachment. It is hoped that through this research, we will develop a deeper understanding of golf leisure activities and provide advice to the relevant industries.

## Literature Review

### Golf-Related Research

In the past, there have been many studies on different market segments, such as seniors or women (Stubbs and MacGregor, 1997; Danylchuk et al., 2015; Kim et al., 2015), and Shoemaker (1989) found that the elderly are not a completely homogeneous market. This means that even among older people, the motivations and reasons for engaging in golf will vary from person to person, thus affecting their leisure activities (Danylchuk et al., 2015). This has deep implications for this study, in that even people of the same age group may have significant differences in their level of activity involvement, and this will have an impact on their subsequent behavior.

In addition, because technology is changing with each passing day and land is expensive in some countries, some countries have begun to develop virtual golf courses, and there has been corresponding academic research (Han et al., 2014). Some countries, such as Cyprus, have come to treat golf as a major business area. Boukas and Ziakas (2013) propose that the golf business is worth investing in as a tourist attraction. There are two main reasons for this. First, the ordinary customers who engage in golf are more likely to be repeat tourists, and the second is because of golf. The balls are targeted more at high-spending groups. From these studies, the importance of golf balls and related research topics can be observed.

Regarding the relationship between activity involvement and local attachment, Lewicka (2011) reviewed nearly 400 related studies published in 120 different journals and identified the importance and intensity of this relationship. Place attachment refers to the relationship between people and specific places (Hwang et al., 2005; Lee and Shen, 2013), usually because of past positive experience, and thus gradually became attached to the place (Kyle et al., 2003). The concept of local attachment is applied in many different fields of research, and the scale of the “place” varies. For example, there is research on a specific restaurant as a subject of local attachment (Debenedetti et al., 2014). From a whole range of research (Ramkissoon, 2015), it can be seen that the application of this concept of local attachment can be carried out in different scales, different industries, and with different variables. Nevertheless, the causal relationship between activities involved in local attachment is still the most commonly used chain of relationships among scholars (Stubbs, 1997; Lewicka, 2011). Therefore, this study also uses this as the infrastructure to construct the framework of this study.

### Hypothesis Development

The relationship between people and places is one of the important topics that academic circles are continuing to explore (Lewicka, 2011). The concept of local attachment was born in, and the concept of local attachment is often applied by, the research institutes of the leisure tourism industry. Most of them are scholars. There is agreement that the degree of involvement associated with the activity is an important pre-institutional factor of local attachment (Kyle et al., 2003). The main concept is that when a person puts more effort into an activity in a place, it will produce stronger feelings, and the feeling of local attachment will then form. This study, therefore, puts forward the following hypothesis:

H1: The “activity involvement” of golf course customers will positively affect their “local attachment.”

The relationship between activity involvement and activity experience stems from the concept of flow (Cheng et al., 2016). Its discussion shows that when a person is fully involved in an activity, it will be awkward. The ecstasy of realm, and thus better experience the fun and benefits of the event. This concept is applied in many leisure activities, especially in serious leisure activities, such as mountaineering (Cheng et al., 2016; Fotiadis et al., 2019), music appreciation or performance (Diaz, 2013; Wrigley and Emmerson, 2013), dance (Thin et al., 2013), etc., so this study proposes the following hypothesis:

H2: The “activity involvement” of golf course customers will positively affect their “activity experience.”

The concepts of customer experience and experience value are derived from marketing (Schmitt and Zarantonello, 2013), mainly for industries that rely on the provision of intangible goods or services, because the customer does not obtain the actual goods, so the goods obtained or the value of the service can depend on subjective experiences. Examples of such industries are wine tours (X. Chen et al., 2016), music festival tours (Andersson et al., 2017), the hot spring industry (Chen et al., 2013), etc. These terms are also used in leisure sports (Cronin and Lowes, 2016). In addition, online shoppers are not able to see the actual physical goods in advance, so these concepts are also used by online merchants for online shopping research. A relationship between the buying experience and its experience value has been explored by Bilgihan et al. (2014), and the current study argues that there is a clear causal relationship between the activity experience and the experience value. The corresponding hypothesis is:

H3: The “activity experience” of golf course customers will positively affect their “experience value.”

The extent that individuals interact with the people, things, or activities provided by a particular place is thought to have a significant impact on the formation of their local attachment (Prayag and Ryan, 2012). Scholars believe that a positive experience can produce unforgettable memories, which in turn add attachment to the place (Huan et al., 2004; Loureiro, 2014; Fotiadis et al., 2016), and even lead to post-event behaviors, such as sharing their experiences with people. For example, the authenticity of a tourist’s experience of a monument will cause local attachment to the monument (Ram et al., 2016). The correlation between experience and local attachment is also applied in sports-related research (Brown et al., 2016), so this study proposes:

H4: The “experience value” of golf course customers will positively affect their “local attachment.”

The degree of activity involvement affects the value of the activity’s own potency (Grohs and Reisinger, 2014). Even when buying physical items, the experience of buying will impact the perception of the value of the actual purchased item (Andrews et al., 2012). From this, it can be seen that the degree of involvement at the time of the event will affect the consumers or the players engaged in the activity, and the value of the goods or services they experience will change. Therefore, this study proposes the following hypothesis:

H5: The “activity involvement” of golf course customers will positively affect their “experience value.”

The degree of involvement in the activity itself also causes people to have deeper feelings about the place in which they do it (Prayag and Ryan, 2012). This kind of emotion is local attachment. Loureiro (2014) argues that local attachment will produce repeat visitations of respondents. When the place has positive behavioral intentions and behaviors associated with it, this leads to a higher estimate of place value and more enjoyable behaviors when in that place (Ram et al., 2016), so this study proposes:

H6: The “activity experience” of golf course customers will positively affect their “local attachment.”

## Methodology

### Research Scope, Objects, and Sampling Methods

This study takes the golf course as an example and uses it as the research scope, while the research object is the golf course customer. The survey was conducted from October 1, 2016, to December 31, 2016. This study used a random sampling method to issue questionnaires and cooperated with personnel to retrieve 534 samples. In terms of gender, most respondents were males, accounting for 60.9%. In terms of age group, “21–30 years old” is the most common, accounting for 42.9%, and “younger than 20 years old” is the second-highest, accounting for 21.3%. It can be seen that there are more and more young people investing in golf. In terms of education level, “university/college” was the most common, accounting for 65.4%, and the “postgraduate or above” was the second-highest, accounting for 21.3%.

### Questionnaire Design

The questionnaire of this study consists of five parts. The main purpose of the first part is to investigate the socio-economic background of the respondents. The other four parts are the four facets of the study, namely activity involvement, activity experience, experience value, and local attachment. In terms of activity involvement, according to the opinions of Funk et al. (2004), it contains three different originals, namely, attraction, self-expression, and life center. For activity experience, according to Han et al. (2014), comments include health environment experience, environmental experience, social experience, and catering-related hospitality services. In terms of experience value, according to the opinions of Chen et al. (2014), customer return on investment, quality of service, pleasure, and beauty are considered. Finally, in terms of local attachment, according to Hwang et al. (2005) and Lee and Shen (2013), local attachment and local identity are considered. Please see the Appendix for the detailed measurement of the questionnaire.

### Data Analysis

To test the hypotheses, the data were analyzed by Amos Software to model the structure and understand the causal relationship between its elements.

To achieve rigor in the structural equation model, first, the reliability and validity of the data were tested. Common Cronbach’s α (Nunnally, 1978), compositional validity, and average extraction variation (Fornell and Larcker, 1981) were calculated and tested.

In order to ensure the compatibility of the model, this study also examined whether the fitness indicators met the standards proposed by other scholars (Hooper et al., 2008; Hair et al., 2009; Kline, 2011). Finally, the overall research structure was analyzed, as was the verification the relevant hypotheses.

## Results

### Reliability and Validity

The reliability and validity indicators related to this study are summarized in Table 1. According to Nunnally (1978), the internal consistency of a Cronbach’s α value above 0.9 is excellent, indicating that the reliability of each facet is sufficient, while 0.7 or above is within an acceptable range but and 0.8 is better.

**TABLE 1:** Reliability and validity.

	α	CR	AVE	MSV	ASV	AE	AI	PA	EV
(1) Activity experience (AE)	0.96	0.97	0.57	0.75	0.49	0.76			
(2) Activity involvement (AI)	0.97	0.95	0.61	0.48	0.38	0.57	0.78		
(3) Place attachment (PA)	0.95	0.95	0.71	0.52	0.47	0.63	0.69	0.84	
(4) Experience value (EV)	0.95	0.95	0.64	0.75	0.53	0.87	0.57	0.72	0.80

*α, Cronbach’s Alpha; CR, composition reliability; AVE, Average Variance Extracted; MSV, Maximum Shared Variance; ASV, Average Shared Variance.*

Fornell and Larcker (1981) suggested that in structural equation model analysis, the composition reliability (CR) of each facet should be better than 0.7, the lowest should be 0.6, and the average extraction variation (Average Variance Extracted, AVE) needs to reach 0.5 to meet an excellent standard is recommended to be at least above 0.36. In addition, the Maximum Shared Variance (MSV) and the Average Shared Variance (ASV) should be as small as possible to ensure the validity level.

According to the analysis results of this study, the Cronbach’s α, CR, and AVE values all have a high quality standard. However, in terms of activity experience and experience value, the MSV is greater than the AVE, indicating that there is a certain degree of commonality between variables or facets. However, this study has reached a high level of indicators, and in non-accurate science, it is difficult to completely discern discriminative problems, so this result has been deemed acceptable for this study.

### Hypothesis Testing Results

**FIGURE 1:**
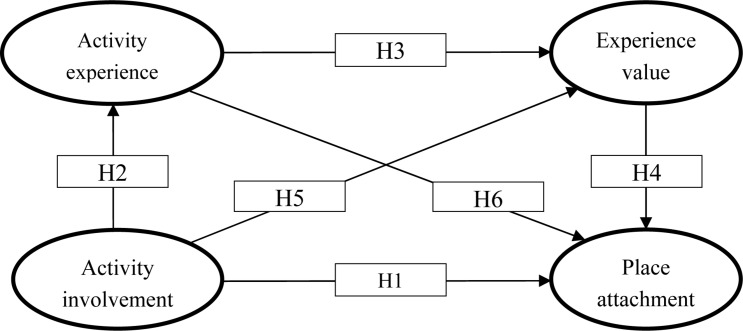
Research model.

**FIGURE 2:**
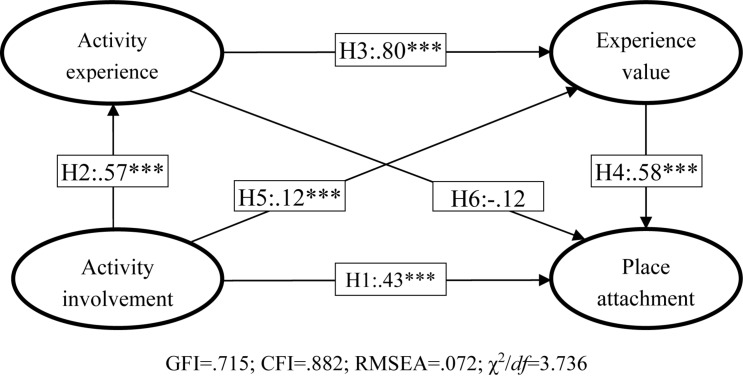
Hypotheses testing results.

The research model with 6 hypotheses is shown in Figure 1 and the model fit index for this study is shown in Figure 2. The model-related indicators are as follows: GFI = 0.715; CFI = 0.882; RMSEA = 0.072; χ^2^/df = 3.736. According to the opinion of many scholars (Hooper et al., 2008; Hair et al., 2009; Kline, 2011), GFI and CFI must be at least 0.7 or higher, RMSEA less than 0.08, and chi-square versus degree of freedom (χ^2^/df) less than 5. The relevant values for this research model are within the acceptable range.

In view of the reliability and validity of the study, the model meets the test criteria, so hypothesis testing was performed. The results are summarized in Table 2 and Figure 2. According to the results, five of the six hypotheses in this study are supported, and the level of significance is less than 0.001. The results are as follows.

**TABLE 2:** Hypothesis testing results.

			Non-standardized coefficient	Standardized coefficient	Standard error	*t*-value	*p*-value
Activity involvement	→	Activity experience	0.43	0.57	0.04	11.86	***
Activity experience	→	Experience value	0.75	0.80	0.05	15.79	***
Activity involvement	→	Experience value	0.08	0.12	0.02	3.67	***
Activity involvement	→	Place attachment	0.33	0.43	0.03	9.89	***
Experience value	→	Place attachment	0.63	0.58	0.08	7.74	***
Activity experience	→	Place attachment	-0.12	-0.12	0.07	-1.68	0.09

***p < 0.001.

H1: The “activity involvement” of golf course customers will positively affect their “place attachment” is supported because the standardized β is 0.43 and the *p*-value is significant.

H2: The “activity involvement” of golf course customers will positively affect their “activity experience” is supported because the standardized β is 0.57 and the *p*-value is significant.

H3: The “activity experience” of golf course customers will positively affect their “experience value” is supported because the standardized β is 0.80 and the *p*-value is significant.

H4: The “experience value” of golf course customers will positively affect their “place attachment” is supported because the standardized β is 0.58 and the *p*-value is significant.

H5: The “activity involvement” of golf course customers will positively affect their “experience value” is supported because the standardized β is 0.12 and the *p*-value is significant.

H6: The “activity experience” of golf course customers will positively affect their “place attachment” is not supported because the *p*-value is 0.09, greater than 0.05.

The model proposed in this study actually has multiple intermediaries.

For example, activity involvement→experience value is mediated through activity experience. The fact that all three paths are significant means that the mediation is only partial at best. The results show that activity involvement is more important in terms of generating good golfing experience and eventually place attachment. The “activity value” plays an intermediary role between “activity experience” and “place attachment”. In other words, the respondents’ “activity experience” does not directly generate their “place attachment,” but it will be indirectly affected through the creation of “experience value.”

## Conclusion and Suggestions

### Conclusion

Along with economic growth, the demands of modern people for quality of life are increasing day by day, and they are pursuing a variety of leisure activities (Wang et al., 2012). Combined with the growth of the aging market (Kim et al., 2015) and of female customers (Danylchuk et al., 2015), this has caused the golf industry to spring up and develop rapidly. Based on this background of earlier research, this study aimed to understand the relationship between activity involvement, activity experience, experience value, and place attachment in golf leisure activities, thereby constructing behavior patterns to provide a reference for operators planning and operating golf courses. Therefore, this study has both theoretical and practical importance.

Based on the above literature review regarding the golf industry, as well as the concepts of activity involvement and place attachment, we know that the golf industry is facing a changing environment, including in business model and in market structure. Understanding the behavior of this market an important goal so as to retain customers and expand to new customers. Therefore, this paper not only has rational meaning in theory but also has practical value for the golf industry.

### Suggestions

In terms of academics: In the past, there were few related literatures and studies on activity involvement, activity experience, experience value, and place attachment in various leisure industries, and there was no in-depth discussion and research on the golf industry and the relationships between the above variables. This study suggests that the research model of this paper be used while adding other variables. The results will establish a more complete consumer behavior model, which will enable the academic community to establish a more complete theoretical foundation and understanding of research regarding golf course customers.

In terms of national development: This study explores the relationship between the activity involvement, activity experience, experience value, and place attachment of golf courses. Therefore, government is recommended to: (1) encourage people to engage in golf activities, (2) promote the advantages of golf to enhance the health of the people and improve their leisure and living standards, (3) encourage entrepreneurs to upgrade the environment and activities at golf courses, and (4) develop and promote the golf industry to enhance the positive image of the city or country.

In other applications: Based on the background of this research, this study reveals the relationship between the activity involvement, activity experience, experience value, and place attachment of golf customers. If this study proves the relationship between activity involvement and place attachment, the research model of this study should also be able to be applied to different leisure or sports industries for planning and management.

## Data Availability Statement

The raw data supporting the conclusions of this article will be made available by the authors, without undue reservation, to any qualified researcher.

## Ethics Statement

Ethical review and approval was not required for the study on human participants in accordance with the local legislation and institutional requirements. Written informed consent from the participants to participate in this study was not required in accordance with the national legislation and the institutional requirements.

## Author Contributions

CC: responsibility of this paper included substantially contributing to developing the research framework, to designing the methodology, to revising the manuscript, and to writing authors’ response notes. S-WL: responsibility of this paper included substantially contributing to designing the methodology, to conducting the survey, to writing, and to searching for references. S-YH and C-HW responsibility of this paper included substantially contributing to conducting the survey, to writing, and to searching for references.

## Conflict of Interest

The authors declare that the research was conducted in the absence of any commercial or financial relationships that could be construed as a potential conflict of interest.

## Appendix

**TABLE A1:** Measure of the questionnaire.

	Section A
1	Playing golf is very fun
2	Playing golf makes me happy
3	I play golf for satisfaction
4	Playing golf keeps me active
5	Playing golf gives me a lot to discuss
6	Playing golf is the focus of my life
7	My relatives and friends often participate in golf
8	I like to talk to friends about the International Music Festival
9	Playing golf is my important way to socialize
10	I have made many golfers
11	Playing golf can relieve stress
12	Playing golf can really be yourself
13	Playing golf shows my values
14	I take my golfing results as my glory
15	I can swing at any time, even without a club

	**Section B**

1	Playing ball can relieve work stress
2	Playing ball helps body coordination
3	When you play well, you have a sense of accomplishment
4	I connect with friends’ emotions through playing
5	Playing golf in Taiwan is a symbol of identity
6	The court has an appropriate level of difficulty
7	Bathroom equipment attached to fairway
8	Spacious and decorated clubhouse
9	Transportation between course fairways
10	Fully computerized course operation
11	Maintenance of golf team safety issues
12	There is good service at the reception counter
13	Course attendant has good service attitude
14	Number of pole brothers
15	Club staff have a good attitude
16	The service staff are very professional
17	Golf club drinks are very good
18	The meal prepared by the golf club was excellent
19	Golf club has well-prepared snacks
20	Golf club has nice cigars or cigarettes
21	Golf club has decent fresh fruit

	**Section C**

1	The quality of service of the golf club is worth my expense
2	Golf club facilities are very new
3	Membership system makes me feel good
4	The service of the staff makes me feel very respected
5	The service of the staff keeps me from worrying about enjoying myself
6	I can have fun every time I come
7	I feel comfortable every time I play
8	Happy playing is really not something money can buy
9	The decoration of the club is very noble
10	The good environment keeps me away from the hustle and bustle
11	The overall environment is very beautiful

	**Section D**

1	I rush to play
2	I feel joy at seeing the perfect arc of the ball
3	I see achievements
4	It has become a habit
5	I have forgotten why I wanted to play golf
6	I feel a sense of achievement in technological progress
7	Playing games makes me feel respect
8	Playing can help me get to know friends
9	I use golf as a fitness activity
10	Playing can make me temporarily forget the hustle and bustle
11	Playing can keep me in touch with friends

	**Section E**

1	I am proud to be a member
2	I want to stay a little longer every time I come
3	The club has become an important place for me to communicate with my friends
4	I often visit golf clubs
5	I like to be better at this golf club than elsewhere
6	This golf club is more important than elsewhere
7	You can get more satisfaction here at this golf club than elsewhere
8	For me, nowhere else can replace this
